# CARA: Connectivity-Aware Relay Algorithm for Multi-Robot Expeditions

**DOI:** 10.3390/s22239042

**Published:** 2022-11-22

**Authors:** Razanne Abu-Aisheh, Francesco Bronzino, Lou Salaün, Thomas Watteyne

**Affiliations:** 1 Nokia Bell Labs, 91620 Nozay, France; 2 Inria, 75012 Paris, France; 3 Univ Lyon, EnsL, UCBL, CNRS, LIP, CEDEX 07, F-69342 Lyon, France

**Keywords:** multi-robot systems, exploration and mapping, relay placement

## Abstract

The exploration of unknown environments is an essential application of multi-robot systems, particularly in critical missions, such as hazard detection and search and rescue. These missions share the need to reach full coverage of the explorable space in the shortest time possible. To minimize the completion time, robots in the fleet must be able to reliably exchange information about the environment with one another. One of the main methods to expand coverage is by placing relays. Existing relay-placement algorithms tend to either require prior knowledge of the environment, or they rely on maintaining specific distances between the relays and the rest of the robots. These approaches lack flexibility and adaptability to the environment. This paper introduces the “Connectivity-Aware Relay Algorithm” (CARA), a dynamic context-aware relay-placement algorithm that does not require any prior knowledge of the environment. We compare CARA against a state-of-the-art distance-based relay-placement algorithm. Our results demonstrate that CARA outperformed the state-of-the-art algorithm in terms of the time to completion by a factor of 10 as it placed, on average, half the number of relays.

## 1. Introduction

The exploration and mapping of unknown environments is an essential application of multi-robot systems, particularly in critical missions, such as hazard detection and search and rescue. These missions share the need to reach full coverage of the explorable space while leaving no parts unexplored [1]. Completeness of the robot-built maps as well as the speed at which this is accomplished are major challenges [2]. Completing the expedition as fast as possible is of high importance, and delays could even imply human losses—for example, in fire detection expeditions.

Communication is one of the most crucial elements of multi-robot coordination, as the efficiency and performance of the mission depends entirely on the real-time data collected by the robots and the timely exchange of it. To minimize the completion time, robots in the fleet must be able to reliably exchange information about the environment with each other with minimum delay. However, in many harsh environments, such as those of critical missions, communications can be delayed, disrupted, or even non-existent due to interference from the environment or from limitations of the robotic platform [3].

In a previous work [4], we demonstrated the need for focusing on communication limitations when designing exploration algorithms by signifying the effect of packet loss on the mapping time to completion. We showed that the higher the packet loss is, the longer the mapping takes to complete. We are therefore interested in methods to compensate for the delay caused by lossy communication when designing and implementing algorithms for multi-robot exploration and mapping.

While not all multi-robot exploration applications require a central base station or “orchestrator”, many do [5]. Having situational awareness at a central orchestrator is often required for the effective supervision of critical missions [6]. When exploring communication-restricted environments, it is essential to maintain reliable connectivity for as far as the robots spread out. We therefore want to expand the exploration range as far as possible, while maintaining connectivity between all robots in the fleet and the orchestrator.

Defining multiple roles (including communication relays) was shown to be a worthy strategy to address this problem [7]. The majority of research on relay placement in such cases tends to fall into two categories. The first is the initial Received Signal Strength Indicator (RSSI)-based communication-aware placement, based on running a full mission prior to the exploration to find the optimal position for the relays to be placed. The second is maintaining a distance (specified prior to the mission) between relays and exploration robots. While these methods do improve the quality of the communication, they add to the time it takes to complete the mission.

Running a separate exploration mission to find the optimal relay position based on building communication models of the area followed by a mapping mission adds significant delay between the moment the fleet of robots is placed in the unknown environment and the moment the map is fully built. Maintaining a certain distance between all robots and all relays requires a high number of relays, which may not all necessarily be needed. This reduces the number of exploration robots, leading to a longer time to completion, given that the higher the number of exploration robots is, the faster the time to completion is.

The main issue at hand is reducing the time to completion, and these methods do not contribute to that. The research question becomes how can we place relays *(i)* to maintain communications as reliably as possible and *(ii)* dynamically throughout the exploration mission without prior knowledge of the environment in a way that minimises both delay to the exploration and mapping time to completion.

As a solution to this problem, we propose Connectivity-Aware Relay Algorithm (CARA), a dynamic context-aware relay-placement algorithm that does not require any prior knowledge of the environment yet positions relays based on the estimated quality of the communications. Communication awareness is when the multi-robot system has the ability to adapt to changes in the quality of communication throughout the mission [8].

Utilising this in relay placement has the benefit of adapting in real-time to the environment the mission is conducted in, to optimise the performance, thereby, making it more flexible to environmental changes. This leads to placing a more optimal number of relays adapted to what is required to maintain reliable communications throughout the mission. By avoiding placing redundant relays, we maximise the number of exploration relays and, hence, reduce the time to completion.

The contributions of the paper are twofold:

- We design CARA, a dynamic relay algorithm that places relays during multi-robot expeditions without prior knowledge of the environment.
- We compare CARA to a state-of-the-art distance-based relay-placement algorithm, demonstrating that connectivity-aware relay placement uses less relays, which, in turn, reduces the time to completion of the multi-robot mission.

The remainder of this article is organized as follows. Section 2 surveys the most relevant related work. Section 3 introduces CARA, our proposed context-aware relay-placement algorithm. Section 4 details the simulation environment and setup used to evaluate CARA. Section 5 describes the simulation results. Finally, Section 6 summarizes the paper and discusses avenues for future work.

## 2. Related Work

There is a multitude of research on how to maintain reliable communications during a multi-robot expedition. However, most of these solutions are not flexible and reactive to changes in the environment in terms of communication, or they require prior knowledge of the environment to some extent.

We classify the related work by answering the following questions:

- Are robots assigned as relays prior to or during the expedition?
- Is any prior knowledge of the environment needed?
- Is the algorithm reactive and adaptable to the quality of the communications?
- Would this algorithm work for multi-robot expeditions that define full coverage of the explorable space as a goal?

### 2.1. General Related Work

Nath et al.  [9] proposed a communication QoS (Quality of Service)-aware A* algorithm to choose the path with the best RSSI out of multiple possible paths. The A* function has an additional metric to the heuristic, which is QoS. Reliable communications is maintained through avoiding paths with poor quality communications and prioritising those with reliable, more stable, communications. However, in exploration and mapping, full coverage of the area is essential; hence, we can not afford to avoid certain paths. We require a solution that improves the quality of communications and increases the coverage throughout the entire explorable area.

Saboia et al.  [10] proposed another communication-aware algorithm that adapts to changes in the dynamic network by using radio propagation models to predict link quality. A connectivity map of the signal quality over the explored area is maintained throughout the expedition. Droppable radios are used as relays, where exploration robots drop these radios in the positions with the lowest Signal-to-Noise Ratio (SNR). This limits speed and flexibility as the relays cannot move themselves, thus, requiring other robots to interrupt their exploration to drop the relay radio at the allocated position.

Kim et al.  [11] proposed a relay-positioning algorithm for multi-agent systems in indoor environments. The robots explore the area of interest prior to the mission to build a communication map using Gaussian-Process-Regression-based link-quality prediction. A heuristic optimization based on Particle Swarm Optimization (PSO) is used to search for the optimal relay positions. Once the optimal relay positions are determined by the optimisation process, the relay agents are dispatched to those positions. While this algorithm may find optimal positions for placing relays, it requires an entire separate mission for placing the relays, as opposed to placing relays throughout the main expedition itself. The number of relay robots appears to be selected prior to the mission.

Gao et al.  [12] proposed a relay control algorithm for end-to-end communication for mobile robots with WiFi routers. They modelled WiFi propagation using a Gaussian Process (GP) to optimally position the relays. However, here also, the relay-placement algorithm is an entire mission of itself, and it is not clear how the number of relays is allocated beforehand. The researchers also depended on prior knowledge of the environment to assist in building their WiFi Propagation model, and this method lacks flexibility and adaptability.

Arnold et al.  [13] used relays to maintain connectivity among a swarm of Autonomous Aerial Vehicles (UAVs). Distance was used as a placement metric. A certain number of UAVs were assigned with the type “Relay” before the mission starts. The role of these Relay UAVs is to maintain a distance equal to half of the maximum range of the WiFi module (approximately 400 m) from the closest member of the swarm. The number of relays—and UAVs that have the role of being relays—is assigned prior to the mission.

Varadharajan et al.  [14,15] proposed an algorithm that creates a chain of relay robots, from a base station to a robot that has lost communication, to “heal” the broken communication in that area. First, a root robot is selected, and then worker robots are selected to perform the task. Once the root and workers are selected, the worker extends the communication chain starting from the root. Then, when a worker has determined that it is a certain distance away from the root it chooses a free robot to be a networker robot to act as a relay to the root.

When that relay is a certain distance away from the root, it chooses another free robot to be an extra relay to maintain a chain of connectivity. This algorithm provides the ability to place the relays dynamically during the mission, which is more advantageous than running a separate mission for relay placement (this strategy requires less time and resources). The algorithm proposed also assigns various roles to the robots during the mission as robots go from being idle (free) to becoming relay robots, as opposed to being pre-assigned as relays prior to the mission. For the reasons mentioned above, we chose this algorithm to compare against our algorithm. We will refer to this algorithm as DBRA (Distance-Based Relay Algorithm) throughout the remainder of this paper to make it easier to reference.

Table 1 summarises the comparison between different state-of-the-art algorithms. From this, we concluded that most existing relay-placement algorithms tend to either require prior knowledge of the environment or rely on maintaining specific distances between the relays and the rest of the robots. This method lacks flexibility and adaptability to the environment. In addition, most works do not consider the time to completion in their relay-placement algorithms.

### 2.2. A Focus on DBRA, the Distance-Based Relay Algorithm

To demonstrate the benefits of CARA, we compare against DBRA [14], a state-of-the-art algorithm that uses distance-based relay placement. Out of all the distance-based work, this algorithm provides the clearest break down as to when, where and which relays are placed, and this makes it the best candidate to replicate and compare against. However, this algorithm was not designed specifically for exploration and mapping. Hence, we made some adjustments to it to help it fit our exploration and mapping target application.

**Figure 1:**
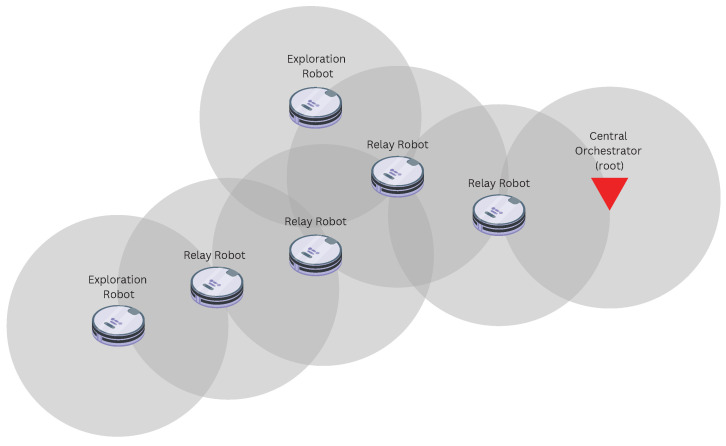
Illustration of a relay tree chain in DBRA.

The objective of DBRA is to construct a tree of relays from a central reference location (i.e., the root of the tree) to the robots that are performing a mission (as illustrated in Figure 1) and have lost connectivity, in order to “heal” broken connections. The distance to be maintained between relays and robots is to be pre-set by a human operator. First, a root robot is selected, and then worker robots are selected to perform the task.

Next, the worker extends the communication chain starting from the root. When a worker has determined that it is beyond the set distance away from the root, it chooses a free robot to act as a relay to the root. When that relay is a certain distance away from the root, it also chooses another free robot to be an extra relay to maintain a chain of connectivity, and so on.

We adjust DBRA to work for exploration and mapping algorithms by making the following adjustments:

- We set the root of the tree chain to be the central orchestrator.
- There are multiple worker robots rather than only one. We consider each exploration robot to be a task robot that needs to maintain connectivity with the orchestrator.
- We have no “free” robots: each robot in the fleet is an exploration robot until it is assigned as a relay when required.
- The original algorithm did not consider obstacles. We adjust the algorithm to take obstacles into account when building the relay chain to, thus, avoid sending a relay to an unreachable position.

Note that these changes do not affect the overall behaviour of the relay-placement algorithm; therefore, they allow for a fair “apples-to-apples” comparison with CARA.

The general behaviour of DBRA is as follows. A distance range is set prior to the mission based on the communications range. This distance can be seen as the radius of a disk around each relaying device, including the orchestrator. All robots start exploring from the location of the orchestrator. Once any robot goes beyond the disk around the orchestrator, a random robot is selected to become a relay and is placed at the boundary of the disk around the orchestrator.

The communication area now becomes the union of the disk around the orchestrator and the disk around the relay robot. Once a robot exits that communication area, another relay is placed in the same manner. What results is a connectivity tree starting from the orchestrator at the root, ending with exploration robots at the edges with relay robots in between.

A limitation of this algorithm is that is does not consider obstacles when choosing the relay position. To address this, we add a mechanism to adjust the relay position if the computed position ends up on an obstacle. A Breadth First Search is ran starting from the chosen position, until the closest explored cell with no obstacles is found. That cell is selected as the relay position, and the relay moves there instead, hence, avoiding obstacles.

## 3. CARA: Connectivity-Aware Relay Algorithm

The aim of CARA is to develop a relay-placement algorithm that can be used along with any multi-robot exploration algorithm, to place relays dynamically throughout the mission, as part of the exploration expedition. The main goal is to minimise the time it takes to complete a multi-robot expedition successfully through the following design choices: *(i)* When to place a relay? *(ii)* Which robot to select as a relay? *(iii)* Where to place that relay? These three choices are essential as they affect both the quality of the communication and the speed of completion.

Two elements that most impact the time to completion in centralised exploration missions are the number of robots exploring, and the quality of communication between the central orchestrator and all the exploration robots. In terms of relay placement, this would translate into the following main requirements: minimising the number of relay robots that switch from exploring the area and become relays that are stationed in specific positions; and maintaining high-quality reliable communications as measured by a certain metric, such as the RSSI or Packet Delivery Ratio (PDR).

Our intuition is that the key to minimising the number of relays is reactivity to the quality of communications in the environment, hence, only placing relays when needed. By avoiding placing redundant relays, we maximise the number of exploration robots, thereby, reducing the time to completion. This is referred to as “communication awareness”: when a multi-robot system has the ability to adapt to changes in the quality of communication throughout the mission.

CARA is a dynamic communication-aware algorithm that does not require any prior knowledge of the environment. It is designed to maintain reliable communication during multi-robot exploration and mapping expeditions to minimise the time to completion. The algorithm dynamically stabilises the communications by keeping the PDR between the orchestrator and robots above a certain threshold throughout the mission. In order to do so, the following is required:

- lowerthreshold: As soon as the PDR of one or more robots goes below this threshold, a new relay must be placed.
- upperthreshold: When the position of the new relay is being chosen, the PDR between the relay and the orchestrator at that position must be above or equal to this threshold.
- CommunicationsHistoryMap: A map that contains the estimated PDR history of every robot in every position it has traversed. This map is updated dynamically throughout the mission

The General behaviour of CARA is as follows. All robots in the fleet start as exploration robots with the role of exploring and mapping the unknown explorable space. They all start exploring from the position of the orchestrator, expanding further away with time. Every 500 ms, all robots transmit a heartbeat packet, thereby, allowing the orchestrator to estimate the Packet Delivery Ratio (PDR).

The orchestrator keeps track of the history of timestamps of when a heartbeat packet was received for each of the robots. Periodically for each robot, the timestamps are used to compute the estimated PDR over a pre-configured sliding window period (in seconds), using (1). For example, if the sliding window period is 10 s, and there are five timestamps stored between t=x and t=x+slidingWindowPeriod, that means that five packets were received, within the last sliding window period, out of an expected 20.

(1) $$estimatedPDR=\frac{numberOfPacketsReceived}{slidingWindowPeriod\cdot 2}$$

The resulting estimated PDR for each robot is stored in the orchestrator’s communications history map along with the position of the robot at the time of estimation. For example: [Robot_id : 5, position : (10.5, 2), estimated_PDR: 0.7]. The orchestrator periodically checks for the estimated PDR values of all the robots. Once the PDR goes below the lower limit threshold for any robot, that robot is set to become a relay robot rather than an exploration robot. Next, the orchestrator chooses a position to send that relay to. It searches the communications history map for the PDR history of the robot and looks for the closest position where the robot had a PDR above the upper limit threshold.

As shown in Algorithm 1, CARA is broken down into four main steps:

(1) Is a relay check due? If the amount of time that has passed since the last check is equal to the sliding window period for checking the PDR, then the answer is yes. Otherwise, do not check if relays are needed yet.
(2) Is a new relay needed? If the estimated PDR is below the lower threshold, then the answer is yes, otherwise no.
(3) Which robot should stop exploring to become a relay? The robot with the PDR below the threshold is the one that should become a relay.
(4) Where should the new relay be sent to be stationed at? This would be the closest position to where that robot currently is that had an estimated PDR above or equal to the upper threshold when it last traversed there.

Based on this, if the PDR never goes below the upper threshold, then no relays will be placed. If it happens once, one relay will be placed, and so on. We do not specify the number or relays prior to the mission, nor do we set which robots are to become relays. Hence, the number of relays placed depends entirely on the quality of communications in the environment, i.e, dynamic context-aware reactivity to the environment.
**Algorithm 1:**CARA algorithm
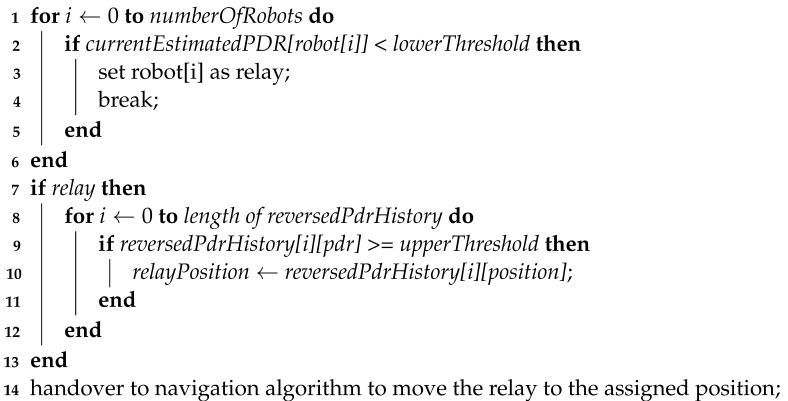


## 4. Simulating CARA

We evaluate the CARA relay placement and compare it to DBRA through simulation. The simulation of both algorithms requires a centralised exploration and mapping algorithm that requires communication between a central orchestrator and all the robots in a fleet as well as a communication model to dictate how the packets are transmitted and received and how we model the quality of communications in an environment.

### 4.1. Setup

We used the Atlas 2.0 exploration and mapping algorithm proposed by Abu-Aisheh et al. [4] to test how CARA affected the performance of the mission verses DBRA. Atlas 2.0 is a centralized algorithm; hence, it relies on robust communication between each robot and the “orchestrator”. Each robot in the fleet receives a new command with movement instructions every time a robot stops upon fulfilling its last given task or bumping into an obstacle. The orchestrator maintains an artificial overlay grid that it builds on the go during the exploration, which can be expanded infinitely. Each grid cell belongs to one of the following categories at every point in time:

- *Explored Open Cells*: Containing no obstacles.
- *Explored Obstacle Cells*: Containing obstacles.
- *Frontier Cells*: The cells surrounding the explored cells that should be explored next to expand the map.

The algorithm is frontier-based, meaning that it moves each robot to a random frontier cell out of the ones that are closest to it. The A* algorithm [16] is used to find the shortest path to the assigned frontier cell. Vectoring is used to break down the new movement instruction for the robot. Vectoring is a navigation service provided to aircraft by air traffic control where the controller decides on a particular airfield traffic pattern for the aircraft to fly. The pattern is composed of specific headings at appropriate timings, and the aircraft follows the headings and timings as instructed by the controller. In Atlas 2.0, the orchestrator has the role of the controller, and the robot replaces the plane.

The movement pattern is the path generated by A*. A robot moves at the speed and in the heading instructed by the orchestrator until its allocated movement duration runs out, unless it encounters an obstacle before then. The overall behaviour is that the frontier expands “away” from the starting position: the robots are coordinated in a way that would “push” the frontier further from the starting point.

In the relay algorithms that we compare, the chosen relay robots will be instructed to move to a “relay position”, as assigned by the algorithms, rather than to a frontier cell. The same navigation method is followed to send the relay from it is current position to it is allocated relay position. The robots navigate and explore in the same manner with both the CARA and DBRA relay placements. The only differences are: (i) when robots are assigned to switch from exploring to becoming relays, (ii) which robots are assigned as relays, and (iii) where the relays are positioned.

**Figure 2:**
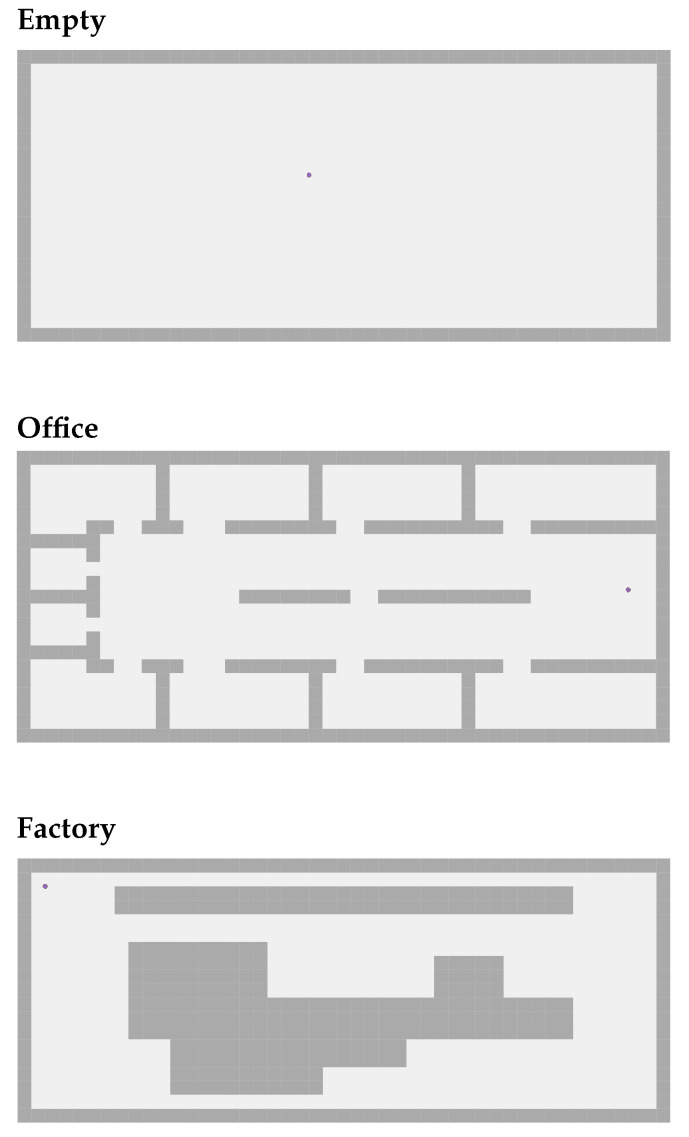
The three simulated floorplans. All floorplan areas are the same size (47 × 21 m${}^{2}$).

To trigger diverse behaviour in the algorithms, we tested them with different floorplans, shown in Figure 2. The “Empty” floorplan is the simplest one: an empty room. We used this as a reference to evaluate the impact of obstacles on the overall performance. The “Office” floorplan represents a more complete end-to-end use case, in which a fleet of robots is tasked to map out a floor of an office building. This could be for a search and rescue mission—for example, to search for victims after a fire or natural disaster. Finally, the “Factory” floorplan is based on a chemical process plant blueprint from [17]. This would be for cases such as hazard detection in a chemical plant.

We also ran simulations with various numbers of robots (15, 25 and 50 robots) to evaluate the impact of the relay-to-exploration robot ratio on the time to completion.

### 4.2. Communication Model

The most crucial element that the relay algorithms depend upon is how the different devices in the system communicate with one another. In this section, we explain the propagation model that we used for the simulations along with the communication protocol.

To compute the link stability directly between any two devices, we use the Pister–Hack (experimental randomness) model [18], which is used to obtain the RSSI between the robots and the orchestrator as shown in Equation (2), where $P_{tx}$ is the transmit power in dBm, $G_{tx}$ and $G_{rx}$ are the transmit and receive gains in dB, *c* is the speed of light in m/s, and *D* is the distance between the transmitter and the receiver in meters. This RSSI value is then translated to a PDR value based on the work done by Municio et al. [19]: We subtract a uniform variance of [0, −40 dB] from the Friis model equation output and convert the RSSI to link stability values using a conversion table based on real-world deployments.

(2) $$\mathrm{RSSI}\left(\mathrm{dBm}\right)=P_{tx}+G_{tx}+G_{rx}+20log_{10}\frac{c}{4\cdot \pi \cdot D\cdot 2.4(\mathrm{GHz})}+rand\left[0,-40\right]\left(\mathrm{dBm}\right)$$

Concurrent Transmissions (CT) refers to tightly synchronized simultaneous transmissions. Multiple nodes in a network transmit the data they want to share with one another simultaneously (within 500 ns). Any nodes that overhear the concurrent/synchronous transmissions receive one of them with a high probability due to the following effects: (i) Capture effect: A receiving radio can capture one of the many colliding packets under specific conditions related to the technology used. (ii) Non-destructive interference: If the colliding packets are tightly synchronized and have the same contents, the resulting signal may be distorted, yet it is highly probable that they will not be destructive to each other. Hence, the receiver can recover the contents with a high probability.

CT embraces the broadcast nature of the wireless medium and synchronizes transmissions to enhance the probability of packet reception. CT also benefits from sender diversity where the concurrent senders have independent links to the receiver. More importantly, this is a simple yet efficient flooding primitive that avoids the implementation and operation overhead of routing and link-based communications. It also achieves enormous performance gains in terms of the end-to-end reliability, latency and energy consumption even under harsh interference conditions [20].

**Figure 3:**
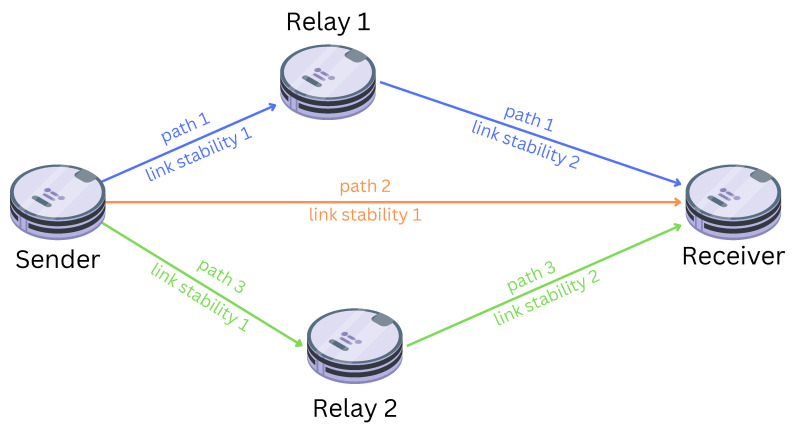
Illustration of concurrent transmissions.

In our model, only the relays re-transmit the packet that they receive simultaneously. If we have no relays, the PDR is equal to the link stability computed based on the RSSI computed using Equation (2). If we have two relays, and one of the exploration robots sends a packet to the orchestrator, as soon as the relays receive that packet, they re-transmit it once. Relay 1 receives packet from Robot x and transmits that exact same packet, and Relay 2 receives the packet from Robot x and transmits the exact same packet. Assuming that the packet reached both relays and that they both transmitted the packet simultaneously within 500 ns of one another, there are now three independent paths through which the orchestrator can receive the packet from Robot x. As shown in Figure 3, these paths are:

(1) Robot x → Relay 1 → Orchestrator.
(2) Robot x → Relay 2 → Orchestrator.
(3) Robot x → Orchestrator.

To compute the total PDR from Robot x to the Orchestrator, we first need to compute the probability of failure for each path, i.e., the probability of the packet not being received at all through that particular path. We apply Equation (3) to all three paths. Then, we compute the total probability of success using Equation (4). The probability of success is the probability of the packet reaching the receiver from the sender.

(3) $$pathProbabilityOfFailure=1-\prod_{1}^{Nlinks}\left(linkStability\right)$$

(4) $$probabilityOfSuccess=1-\prod_{1}^{Npaths}\left(pathProbabilityOfFailure\right)$$

## 5. Simulation Results

We used the Atlas open source simulator developed by Abu-Aisheh et al. [4] to simulate 1800 exploration and mapping runs in total, including the three floorplans as well as three different fleet sizes. (As an online addition to this article, all the source code used is published under an open-source license at https://github.com/openwsn-berkeley/Atlas (accessed on 11 November 2022)

We ran the CARA simulations with an upper threshold of 0.9 and a lower threshold of 0.8. The distance specified for the DBRA disk range was 7 m. The work of Brun et al. [21] demonstrates how, due to multi-path fading, PDR can not be directly mapped to distance at all times. Therefore, the distance of 7 m was chosen based on trial and error to determine the optimal distance where robots did not lose connectivity completely while minimising the number of relays placed.

In this section, we evaluate and compare CARA against DBRA in terms of the time to completion, number of relays placed and PDR evolution as relays are placed. All results are presented with 95% confidence intervals.

### 5.1. Impact of Relays on Time to Completion

**Figure 4:**
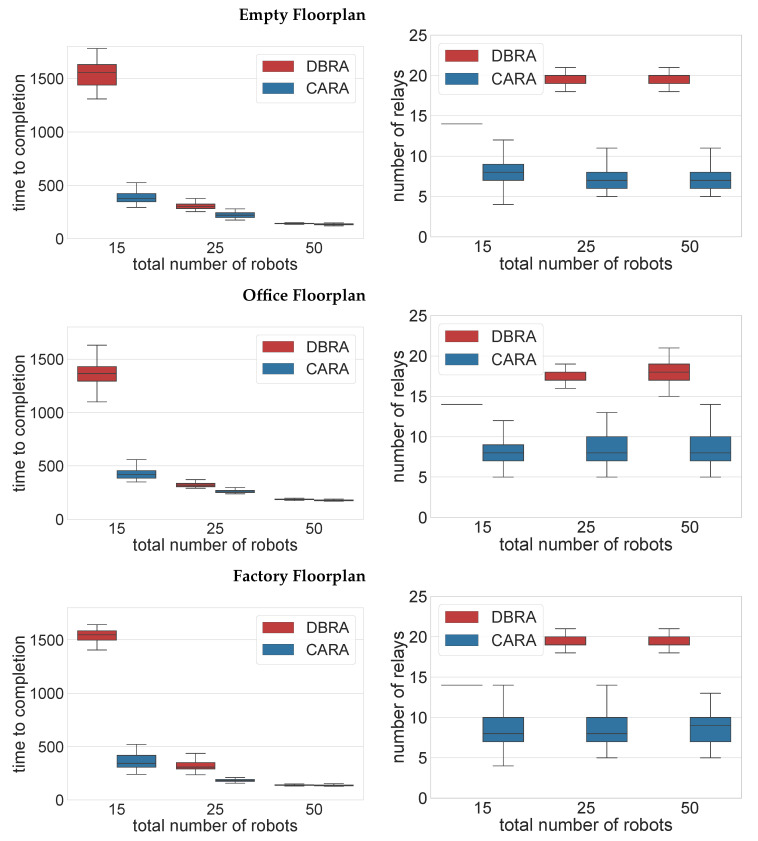
The time to completion and the number of relays placed by the end of the mission for all floorplans with three different fleet sizes.

Figure 4 shows that DBRA takes almost 10-times longer to complete the exploration with 15 robots. The higher the number of robots in the fleet, the smaller the difference in the time to completion between both algorithms. This is due to the ratio between the relay robots and the exploration robots.

With 15 robots, CARA places, on average, eight relays by the end of the mission, whereas DBRA places 14 consistently in every case (as there is no variance, the “box” appears as a line in the plot). CARA places approximately 50% of the fleet as relays while DBRA place 90%, which is a drastic difference, leading to a significant difference in the time to completion. Consequently, DRBA will only have 10% of the fleet exploring towards the end of the exploration compared to CARA with 50%. With 50 robots, CARA places 16% of the fleet as relays, and DRBA places 40%, leaving DRBA with 60% of the fleet to explore even after all relays are placed. Similar behaviours can be observed for all three floorplans.

Figure 4 also demonstrates how CARA is more reactive in terms of the number of relays placed. With 15 robots, DBRA almost always ends up placing 14 relays by the end of the mission, whereas, with CARA, it is somewhere between 5 and 12. This shows that CARA does not always place the same number of relays. The number of relays placed varies based on the PDR, which is the only difference between all the simulation runs with the same floorplan and number of robots. Similar behaviour is observed with different fleet sizes and floorplans.

### 5.2. Evolution of PDR as Relays Are Placed

**Figure 5:**
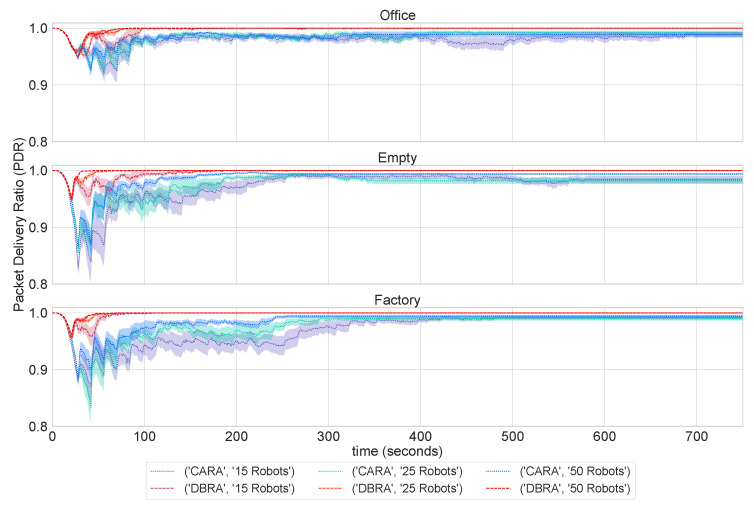
Evolution of the average PDR over time for all floorplans. After 750 s, the data remains stable for the remainder of the experiment.

Figure 5 shows the evolution of the average PDR of all the robots with time. We see that, with CARA, the PDR does indeed remain above the upper threshold of PDR = 0.8 at all times and for all configurations. However, DBRA maintains a PDR of almost 1 at all times. This means that DBRA will loose less packets compared to CARA.

Thus, with CARA, a packet may take longer to reach the receiver, which, in turn, adds delay to the overall expedition. However, given that CARA completes the exploration 10-times faster than DBRA, we conclude that the impact of the number of relays on the time to completion is far more significant than the 10% difference in the average PDR as long as communications is assured throughout the mission assuring no loss of data.

**Figure 6:**
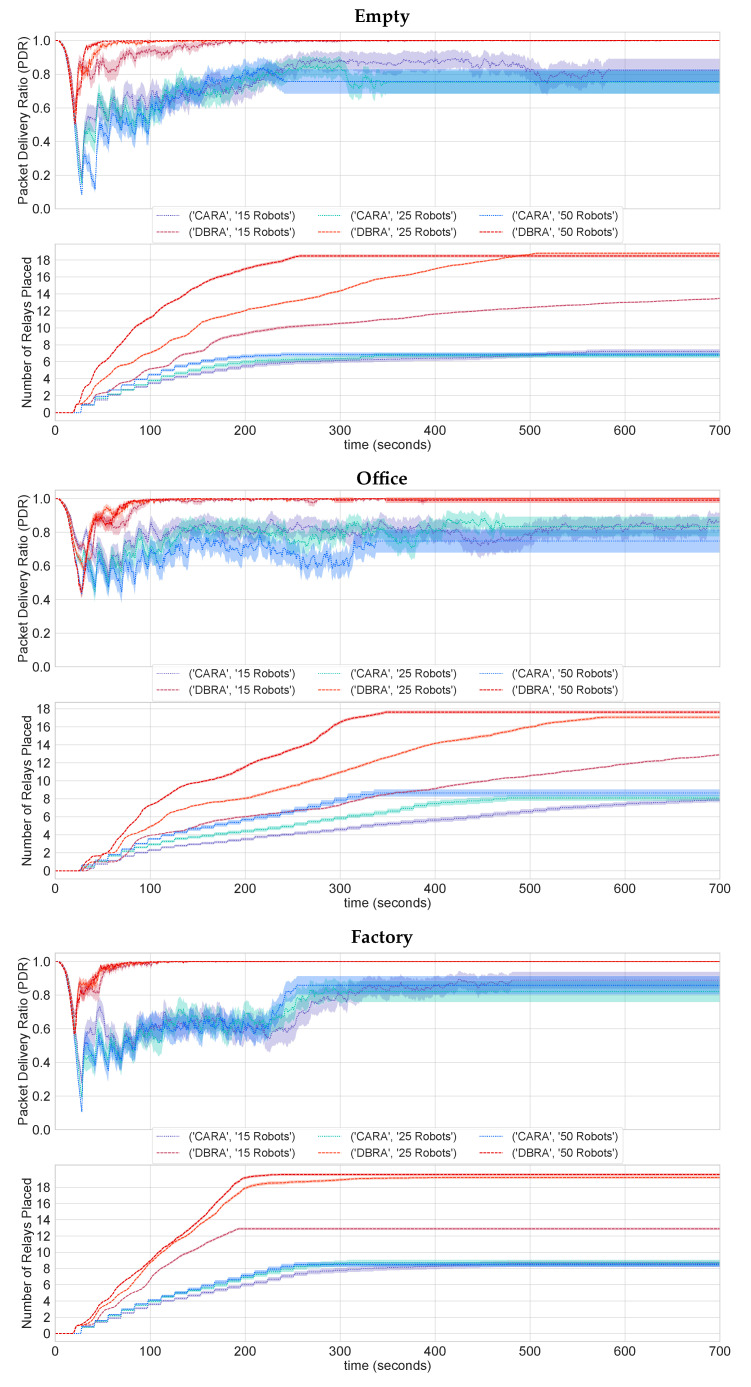
Evolution of the minimum PDR as relays are placed for all floorplans. After 700 s, the data remains stable for the rest of the experiment.

Figure 6 shows the evolution of the minimum PDR with time. We see how the PDR continues deteriorating until the first relay is placed for both algorithms. From there, we see how the minimum relay spikes back up. Here lies the difference in both algorithms. With CARA, we can map the change in PDR to the relay placement. We see that, as the minimum PDR dips below the lower threshold, a new relay is placed, and then the PDR begins to rise again. On the other hand, with DBRA, after around 100 s, the PDR appears to be stable at 1 with eight relays in place. Yet, more relays continue being placed, despite not being needed, which unnecessarily adds to the overall time to completion.

In general, we see how adding communication awareness optimises the number of relays placed. The challenge mostly lies in balancing between reliable communications, represented in our work in terms of the PDR, and between the time to completion. CARA is a step towards this balance, as it maintains an average PDR above 0.8 at all times with different floorplans and fleet sizes. It also completes the exploration 10-times faster than DBRA despite the fact that DBRA maintains a 10% higher average PDR throughout the mission.

## 6. Conclusions

The exploration of unknown environments is an essential application of multi-robot systems, particularly in critical missions, such as hazard detection and search and rescue. These missions share the need to reach full coverage of the explorable space in the shortest time possible. To minimize the completion time, robots in the fleet must be able to reliably exchange information about the environment with each other. One of the main methods to expand coverage is by placing relays.

However, existing relay-placement algorithms tend to either require prior knowledge of the environment or to rely on maintaining specific distances between the relays and the rest of the robots. This lacks flexibility and adaptability to the environment. We focused on the research question of how we can place relays *(i)* to maintain communications as reliably as possible and *(ii)* dynamically throughout the exploration mission without prior knowledge of the environment in a way that minimises both delay to the exploration and the mapping time to completion.

This paper introduced CARA (Connectivity-Aware Relay Algorithm), a dynamic context-aware relay-placement algorithm that does not require any prior knowledge of the environment. We evaluated CARA against a state-of-the-art distance-based algorithm to compare the connectivity performance and time to completion. Our results demonstrated that CARA outperformed DBRA in terms of the time to completion by a factor of 10 as it placed, on average, half the number of relays that DBRA did by the end of a mission.

Future work includes testing CARA on a physical multi-robot testbed to evaluate how the algorithm works under real-life constraints. The next steps include improving the algorithm to consider further contextual physical constraints (such as localisation errors) to make CARA more dynamic and adaptive.

## Figures and Tables

**Table 1:** A comparison between state-of-the-art algorithms for maintaining reliable communications in multi-robot applications.

	When Are Relays Assigned	Prior Knowledge Required	Communication Awareness	Suitable for Exploration
Nath et al. [9]	No relays placed	✓	✓	✗
Saboia et al. [10]	During exploration	✓	✓	✓
Kim et al. [11]	Prior to exploration	✓	✓	✓
Gao et al. [12]	Prior to exploration	✓	✓	✓
Arnold et al. [13]	Prior to exploration	✗	✗	✓
Varadharajan et al. [14]	During exploration	✗	✗	✓

## Data Availability

Not applicable.
